# 3D-Printed electrochemical sensor-integrated transwell systems

**DOI:** 10.1038/s41378-020-00208-z

**Published:** 2020-10-05

**Authors:** Pradeep Ramiah Rajasekaran, Ashley Augustiny Chapin, David N. Quan, Jens Herberholz, William E. Bentley, Reza Ghodssi

**Affiliations:** 1 Institute for Systems Research, University of Maryland, College Park, MD USA; 2 Fischell Department of Bioengineering, University of Maryland, College Park, MD USA; 3 Department of Psychology and Neuroscience and Cognitive Science Program, University of Maryland, College Park, MD USA; 4 Institute for Bioscience and Biotechnology Research, University of Maryland, College Park, MD USA; 5 Robert E. Fischell Institute for Biomedical Devices, University of Maryland, College Park, MD USA; 6 Department of Electrical and Computer Engineering, University of Maryland, College Park, MD USA

**Keywords:** Electrical and electronic engineering, Chemistry

## Abstract

This work presents a 3D-printed, modular, electrochemical sensor-integrated transwell system for monitoring cellular and molecular events in situ without sample extraction or microfluidics-assisted downstream omics. Simple additive manufacturing techniques such as 3D printing, shadow masking, and molding are used to fabricate this modular system, which is autoclavable, biocompatible, and designed to operate following standard operating protocols (SOPs) of cellular biology. Integral to the platform is a flexible porous membrane, which is used as a cell culture substrate similarly to a commercial transwell insert. Multimodal electrochemical sensors fabricated on the membrane allow direct access to cells and their products. A pair of gold electrodes on the top side of the membrane measures impedance over the course of cell attachment and growth, characterized by an exponential decrease (~160% at 10 Hz) due to an increase in the double layer capacitance from secreted extracellular matrix (ECM) proteins. Cyclic voltammetry (CV) sensor electrodes, fabricated on the bottom side of the membrane, enable sensing of molecular release at the site of cell culture without the need for downstream fluidics. Real-time detection of ferrocene dimethanol injection across the membrane showed a three order-of-magnitude higher signal at the membrane than in the bulk media after reaching equilibrium. This modular sensor-integrated transwell system allows unprecedented direct, real-time, and noninvasive access to physical and biochemical information, which cannot be obtained in a conventional transwell system.

## Sensors: Electrochemical measurements of cultured cells

Researchers in the United States have developed a novel system which can measure cellular and molecular events in cell cultures non-invasively and in real-time. The new approach builds on transwell system and is designed to be compatible with existing protocols and tools. The team, led by Reza Ghodssi of the University of Maryland, added electrochemical sensors to the porous membrane normally found in transwell systems, enabling them to measure characteristics of the cultures. An impedance sensor is used to measure cell attachment and growth, while cyclic voltammetry sensors at the bottom measure molecular concentration. The modular platform is 3D printed from autoclavable materials, making the system reusable. With this tool, researchers will have unprecedented access to study the cellular and molecular activity in cell cultures.

## Introduction

Transwell inserts^1^ are one of the most widely used tissue culture platforms to study barrier formation^2^, drug delivery^3^, cell migration^4^, and cell invasion^5^. Commercial transwell architectures are characterized by a porous cell culture membrane suspended between two chambers of media^1^. This substrate is often used to mimic the flexible and permeable in vivo basement membrane, which is composed of protein fibers through which molecular signal transduction occurs^6^. Understanding such inter- and intracellular signaling is the overarching goal of most bioanalytical systems^7^. Platforms for studying dynamic intercellular signal transduction require interfaces supporting cells/tissues and relevant sensing modalities^8–10^. These signal dynamics are often difficult to detect in a transwell or other standard cell culture platforms due to the lack of interfacial and integrated real-time sensing modalities. Though transwell systems have been used extensively over many decades, they have undergone minimal customization both in academic^11–13^ and commercial settings (Snapwell^14^ and Netwell^15^). Most omics (genomics^16^, transcriptomics^17^, proteomics^18^, lipidomics^19^, and metabolomics^20^), histology^21^, and sensing in transwell systems are carried out with external probes ex situ or postmortem. This process requires several steps, during which temporal resolution and molecular information from the signal transduction occurring at the cellular interface may be lost^22,23^. Thus, there is a tremendous need for sensor-integrated cell culture platforms with built-in real-time molecular sensing capabilities.

Two of the most customizable and high-throughput sensing modalities used for collecting cellular and molecular information from cell cultures are electric cell-substrate impedance sensing (ECIS)^24^ and cyclic voltammetry (CV)^25^. ECIS can give extensive information on physical phenomena such as cell attachment^26^, proliferation^27^, migration^28^, differentiation^29^, inflammation^30^, and invasion^31^. This technique requires interfacing cells with a pair of inert metallic electrodes, to which varying frequencies of a sinusoidal AC signal are applied. The resulting impedance and phase responses at characteristic frequencies can provide information related to the aforementioned physical phenomena. Meanwhile, CV can be used for qualitative and quantitative molecular sensing. Previously, CV electrodes have been interfaced with transwell-type systems via either direct fabrication in downstream microfluidic compartments^32^ or by insertion of external microfabricated CV probes^22^ to facilitate real-time and label-free biomolecular sensing. More recently, a miniaturized CV electrode in a 3D-printed microdevice was used to sense mammalian cell culture metabolites^33^. However, as some of these probes are located at a significant distance from the cells or tissues of interest, the metabolites may become diluted or broken down before they reach the CV probe for sensing and detection.

Integrating versatile and well-studied physical and chemical signal transduction elements, such as impedance and CV electrodes, into a transwell membrane can provide ample real-time data on several cooccurring biophysical and biochemical phenomena, advancing research in cellular and molecular biology. Motivated by this need, here, we present the design, fabrication, and testing of a biocompatible multimodal sensor-integrated transwell cell culture platform featuring (i) a porous membrane, analogous to those present in transwell systems, (ii) cell-interfaced Au impedimetric sensors integrated on the top side of the membrane to noninvasively monitor cell culture confluence, (iii) membrane-integrated CV sensors on the bottom side of the membrane with Au counter/working and Ag/AgCl reference electrodes to perform molecular sensing, and (iv) a 3D-printed and sterile housing that supports this membrane and transepithelial electrical resistance (TEER) probe access as a standard measure of cell layer confluence.

We demonstrated (i) culturing and impedimetric sensing of a triculture of epithelial cell lines—including Caco-2, HT29-MTX, and RIN14B cells—and (ii) quantitative and qualitative dynamic molecular sensing of ferrocene dimethanol (FDM) as it was injected above the membrane and diffused to the CV sensors at the bottom of the membrane, mimicking a metabolite release event from the basolateral (bottom) side of a cultured cell layer. The sensors were fabricated and tested individually (membranes with only interdigitated impedance electrodes or only CV electrodes) and together (devices in which impedimetric and CV electrodes are fabricated back to back on the same membrane) to realize a multimodal sensor-integrated transwell system. The design, mode of assembly, and choice of materials enable measurements to be taken inside an incubator or on a benchtop without compromising the sterility of its internal contents.

Integrating the sensors onto a porous cell culture membrane and packaging it within a 3D-printed cell culture platform imparts real-time in situ sensing capability within this cell culture system. The use of simple additive manufacturing techniques enables customizable and modular designs that are autoclavable and reusable, circumventing conventional microfabrication processes. The combination of features offered by this platform enables its use as a discovery tool and in lieu of a conventional transwell in cellular/molecular biology.

## Results and discussion

### Fabrication of a sensor-integrated membrane

The dimensions of the sensor-integrated membrane (diameter: 15 mm) are chosen to closely match the dimensions of a single well of a 12-well transwell insert (diameter: 15.6 mm) to minimize deviations from standard operating protocols (SOPs) of cell culture. Microporous polyester track-etched (PETE) membranes were used as the substrate for sensor fabrication. The electrodes on the membranes were fabricated with e-beam evaporation through laser-cut paper shadow masks. Standard microfabrication techniques such as photolithography were not used, as this process would expose the cell culture membrane to cytotoxic organic solvents, compromising the biocompatibility of the system^34^. A pair of Au electrodes was used for impedance measurements, and a three-electrode system consisting of Au working, Au counter, and Ag/AgCl reference electrodes was used for potentiometric measurements. Two modules of electrode designs are considered. Case I: the impedance electrodes and the CV electrodes were fabricated on two different membranes, and case II: the impedance and the CV electrodes were fabricated back to back on the same membrane for multimodal sensing.

### Case I

**Fig. 1:**
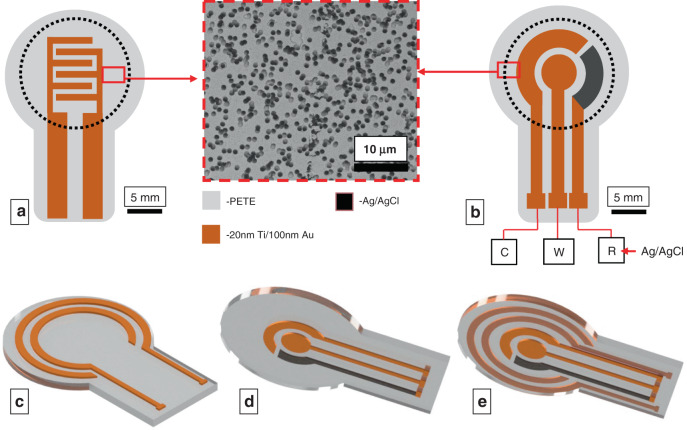
Electrode-integrated membrane designs. Case I: **a**, **b** Schematics of **a** impedance interdigitated electrodes (IDEs) and **b** circular CV electrodes fabricated separately on two different PETE membranes. The image at the center shows an SEM image of the Au-coated membrane, showing that the track-etched pores are open even after Au deposition. The pore diameter is 1µm, and the porosity is 2×10^7^ pores/cm^2^. C, W and R represent the counter, working, and reference electrodes, respectively. Case II: **c**–**e** Schematics of the **c** concentric impedance electrodes on the top side of the membrane, **d** CV electrodes on the bottom side, and **e** integrated arrangement of both impedance and CV electrodes on one membrane. The rectangular trace in all electrodes represents the contact pads that protrude outside the sensing area of the platform

A pair of impedance electrodes was patterned in the form of interdigitated electrodes (IDEs) on the top side of the membrane, on which the cells can be grown directly. The CV electrodes do not come in direct contact with the cells since the high current generated during CV can be potentially lethal to the cells^35^. Hence, the CV three-electrode system was fabricated on the bottom side of the membrane, where the cells are insulated from the electrodes (Fig. 1a, b). The leads of both sensors are extended to serve as contact pads.

### Case II

To demonstrate multimodal sensing functionality, impedance and CV electrodes were fabricated on opposite faces of the same membrane. In lieu of IDEs patterned at the center as in case I, a pair of concentric circular impedance electrodes was fabricated on the top side of the membrane to avoid spatial overlap and electrical shorting with CV electrodes, as metal deposition through the pores may connect the top and bottom electrodes (Fig. 1c–e).

### Development of sensor-integrated cell culture housing

**Fig. 2:**
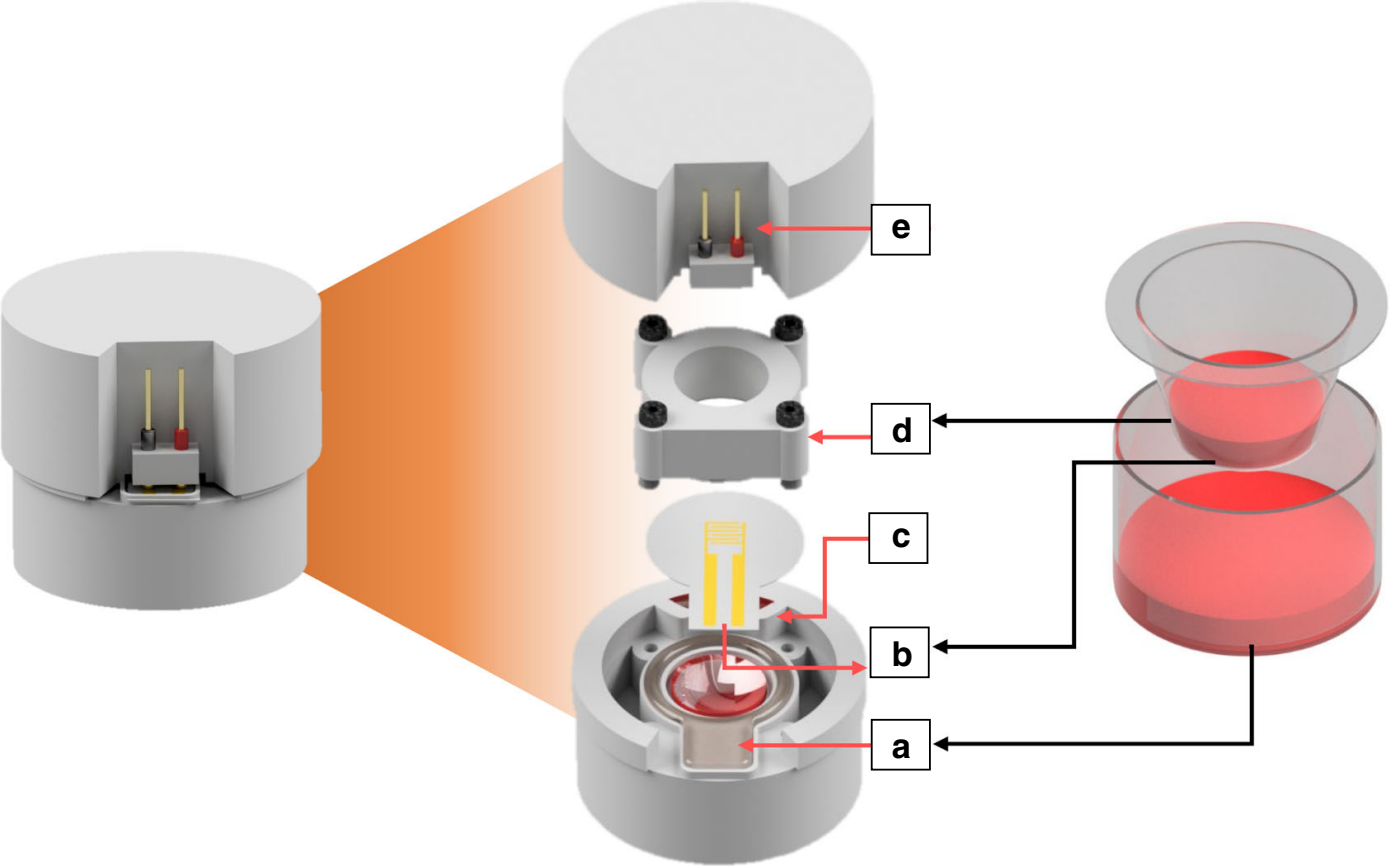
3D-printed transwell platform design. A side-by-side comparison of our sensor-integrated 3D-printed transwell (left) and a commercial transwell (right). (Left) Illustration showing the exploded view of the 3D-printed sensor-integrated cell culture platform, with labeled components: **a** 3D-printed bottom chamber filled with cell media (red) and in situ PDMS gaskets for membrane attachment, **b** electrode-integrated PETE membrane with contact pads, **c** access port to provide fluid to the bottom chamber and serving as an accessibility point for TEER electrodes, (see also Supplementary Fig. S1), **d** top chamber, and **e** electrodes to connect to the sensor contact pads. Analogous parts are labeled in the transwell on the right

Figure 2 shows a schematic of the sensor-integrated 3D-printed transwell in comparison to a commercial transwell. Unlike that of commercial transwells, the configuration of the 3D-printed transwell housing is not concentric (well-in-well) but stacked (well-on-well) (Fig. 2). The stacked design was adopted to provide a planar cross section for electrical access to the sensors via the electrode leads, which would be more difficult in a traditional transwell. This architecture is achieved by sandwiching the electrode-integrated membrane between the top and bottom chambers, attaching via in situ fabricated PDMS gaskets (Fig. 2a, b, d), and tightening with metric screws. The elastomeric nature of the in situ PDMS gaskets prevents the electrodes from fracturing under tightening force while simultaneously preventing leaking. A fluidic access port is included to fill the bottom chamber with media (Figs. 2c and Supplementary Fig. S1a). This port is designed to be filled to above the level of the membrane, providing a positive pressure to keep the media in contact with the membrane, as in a commercial transwell. This port also provides access to the bottom chamber media, facilitating TEER measurements with a standard TEER probe (Supplementary Fig. S1b).

The completely assembled system with all the components (except the spring-loaded pins and the holder, Fig. 2e) is autoclaved at 121 °C for 30 min to sterilize before use. After assembly, ohmic contact is established between the sensor contact pads on the membrane and the spring-loaded pins outside the lid (Fig. 2e) for electrochemical measurements.

### Impedance sensing

Following assembly and sterilization, the sensor-integrated 3D-printed transwell chambers are filled with cell culture media. The device is incubated for 24 h to passivate the device and leach out any residual chemicals from the 3D printing process. This process will also coat the electrode-integrated membrane and the entire platform with nonspecifically adsorbed proteins from the media, promoting cell adhesion and enhancing the biocompatibility of the platform^36^. After 24 h, fresh media is added to the bottom and top chambers before seeding the platform with cells. A triculture of Caco-2, HT29-MTX, and RIN14B cells in the ratio 4:2:1 is seeded on the electrode-integrated membrane 3D-printed within the platform: (1) *Caco-2* enterocytes form tight junctions that hold together all of the other cell types; (2) *HT29-MTX* goblet cells produce mucus, the foremost protective layer covering the gut from mouth to colon; and (3) *RIN14B* model enterochromaffin cells (ECCs) release serotonin upon luminal stimulation with various chemical, physical, and osmotic conditions^37^. These cells were chosen to represent specific functions of the mammalian gut epithelium, such as barrier formation and molecular release. The ratio of cells in triculture was chosen to reflect their in vivo distribution (Caco-2 ≫ HT29-MTX > RIN14B) and to recapitulate relevant functional features given this set of model cells^38^.

**Fig. 3:**
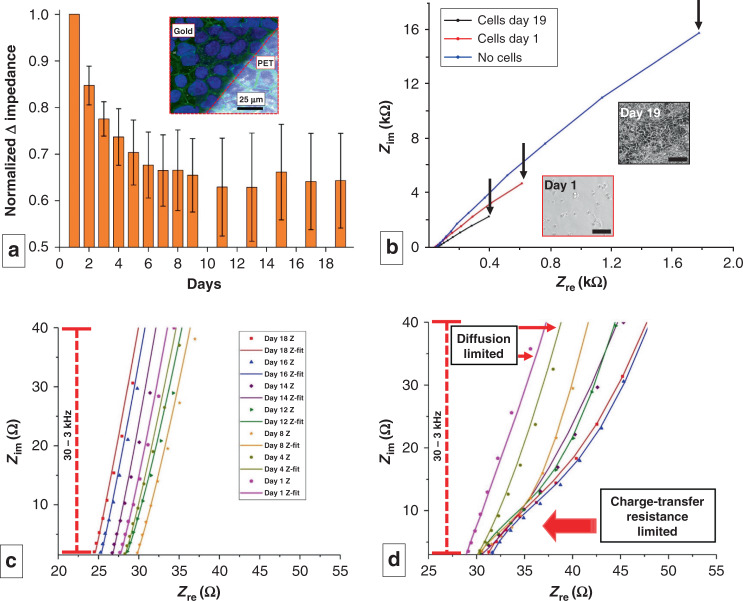
Impedimetric analysis of cell culture on IDEs. **a** Normalized change in impedance values measured at 10 Hz over the course of 19 days (*n* = 3 measurements from separate devices). The inset shows the endpoint (day 19) fluorescence micrograph of the cell-covered sensor surface superimposed on the brightfield image of the sensor. The dark area denotes the area covered by the Au electrode, and the bright area corresponds to PETE. **b** Nyquist plot showing the impedance at 3 stages of cell culture. The black arrows indicate impedance at 10 Hz. Insets show the phase contrast image of the triculture on day 1 and day 19 (Scale bar: 100 µm). **c**, **d** Zoomed in Nyquist plots for **c** control device with no cells and **d** device with cell culture. The latter indicates the transition of the system from a diffusion-limited state to a charge transfer-limited state as the culture progresses. (dots = raw data, line = fitting with model on Supplementary Fig. S2). **c**, **d** Share the same legend

Figure 3a shows the monitoring of cell culture growth via impedance and a representative fluorescence microscopic image of the final culture. The impedance of the cells cultured on this platform was measured at 24 h intervals for 19 days until the impedance started to plateau. Impedance values derived from the Bode plot indicate that the normalized change in impedance at 10 Hz decreases exponentially and stabilizes over the time course of the cell culture (Fig. 3a) (*n* = 3 measurements from separate devices). This behavior correlates with the initial log phase, where the cells grow exponentially, followed by a stationary phase, where the cells become confluent and stop multiplying^39^. The absolute impedance decreased by an average value of 160% from day 1 to day 19. As shown by our previous work, the observed decrease in impedance corresponds to the increased capacitance due to ion accumulation from the cell-associated extracellular matrix (ECM) on the surface of the electrode^40–42^. The ECM is composed of mostly protein and DNA, which are charged molecules that can increase the dielectric permittivity of the interface between the electrode and media. This increased dielectric permittivity increases the double layer (Debye layer) capacitance, which, in turn, decreases the impedance in the low-frequency range. To further ascertain whether this drop in impedance is due to the accumulation of double layer ions within the ECM, the surface of the sensor was covered with analogous microparticles (alumina powder with an average particle size of 1 μm) dispersed in Dulbecco’s modified Eagle medium with 10% fetal bovine serum (DMEM + FBS). Cultured mammalian cells will secrete ECM over time, creating a layer that can reach thicknesses of 1–10 µm^43^ wherein the Debye length is on the order of the relevant microstructures of the ECM^44^. Alumina (Al_2_O_3_) beads, on the other hand, cannot produce ECM with time; thus, their Debye layer will only be a few nanometers thick^45^ and will not change significantly over time. Hence, we used this material as a negative control for comparison to the cell growth conditions used in our experiment; alumina beads with a 1 µm diameter were chosen for their ability to cover the surface efficiently with the desired packing efficiency. Particles much smaller than 1 µm may go through the pores, as the pores are approximately 1 µm, and larger particles may leave voids while packing on the surface, through which the electrolyte will directly come in contact with the electrode. While ~100% coverage of alumina on the membrane showed an ~7% decrease in impedance, ~100% coverage of cells showed a 68% decrease in impedance, as indicated in Supplementary Fig. S3.

The superimposed confocal and brightfield image of the membrane shows that the cells form a confluent monolayer across the sensor surface (PETE-Au interface) within the 3D-printed platform (Fig. 3a, inset). The area covered by Au appears darker, as it is opaque in transmission-mode brightfield microscopy. Blue and green fluorescence represent DAPI-stained nuclei and tight junctions (occludin), respectively. Immunocytochemistry of the triculture grown on the membrane validates cell viability and biocompatibility of the 3D-printed sensor-integrated platform, enabling the potential use of this platform for different molecular biology and biotechnology studies.

Figure 3b shows the corresponding Nyquist plots measured at three time-points of cell culture in one device: (i) no cells (bare electrode), (ii) day 1 (seeding), and (iii) day 19 (confluent monolayer). As shown, there is a drop in both the real and imaginary impedance at 10 Hz (indicated by arrows), which is dominated by the double layer capacitance of the ECM-covered electrode-cell interface. The inset shows a phase contrast image taken on days 1 and 19 of a representative triculture plated under identical conditions on a 12-well plate, as the cells grown on the membrane cannot be imaged via transmission-mode brightfield microscopy due to the opacity of the cell culture membrane. An identical number of cells were plated on the device and 12-well plate and imaged during every impedance measurement of the device to corroborate the measured impedance with the cell growth under identical conditions. Though the cell behavior may be different on PETE membranes vs. polystyrene plates, we anticipate that the images taken in the plates are representative of the cell growth on the device, as we have already accomplished triculture growth and tight junction formations on the electrode-integrated membrane (Fig. 3a, inset).

Figure 3c presents the Nyquist plot of the control where no cells were seeded. The linear trend between the real and imaginary parts of the impedance is attributed to diffusion-limited charge transfer kinetics^46^ associated with the no-cell condition. In contrast, in Fig. 3d, where the cells adhere and cover the electrode surface, the Nyquist plot shows the development of a suppressed semicircle that is characteristic of the emergence of a charge transfer resistance-limited state^46^. In other words, we hypothesize that the tightly packed monolayer of cells covering the electrode may resist charge transfer between the electrode and the media. This hypothesis is also corroborated by the TEER values measured with the conventional TEER electrodes on days 14, 16, and 18, which were 708 Ω/cm^2^, 1105 Ω/cm^2^, and 1615 Ω/cm^2^, respectively. These values correspond to the formation of tight junctions in the 3D-printed culture platform^23^.

### Electrochemical sensing with cyclic voltammetry (CV)

While impedance sensing can be used to noninvasively investigate a plethora of physical phenomena, electrochemical CV sensors directly fabricated on the tissue culture membrane can monitor other characteristics of the cell culture, such as temporal dynamics and concentrations of specific metabolites and biomarkers of interest. Of particular interest is to characterize the detection of molecules in real time as they are released from the cell layer and diffuse through the porous membrane to react at the CV electrodes as a mechanism for cell metabolite detection. For example, the redox molecule serotonin is present in abundance in the gut and is hypothesized to be modulated by the gut microbiome^47^. Electrode-integrated cell culture platforms, such as the one demonstrated here, could be potentially customized to sense the temporal release of electrochemical molecules, such as serotonin, from an in vitro gut tissue model^48^. Further, we theorize that, given the ability of CV to detect a wide range of biomolecules, our device could be modified in future work to perform global metabolic profiling^49,50^. For example, CV has been used to detect many types of redox-active molecules and ROS^51^, including many neurotransmitters such as serotonin, dopamine, melatonin, and norepinephrine^52,53^. Other non-redox-active molecules have also been detected by modifying CV electrodes with transducing elements such as cell-based sensors^54^, enzymes or aptamers so that non-redox-active molecules such as glucose^55^, albumin^32^, and alcohols^56^ can be quantified. The ability to adopt these sensing modalities would be predicated on the development of sensors with the appropriate specificity to each analyte and the ability to arrange them in a compact array on the porous membrane surface.

**Fig. 4:**
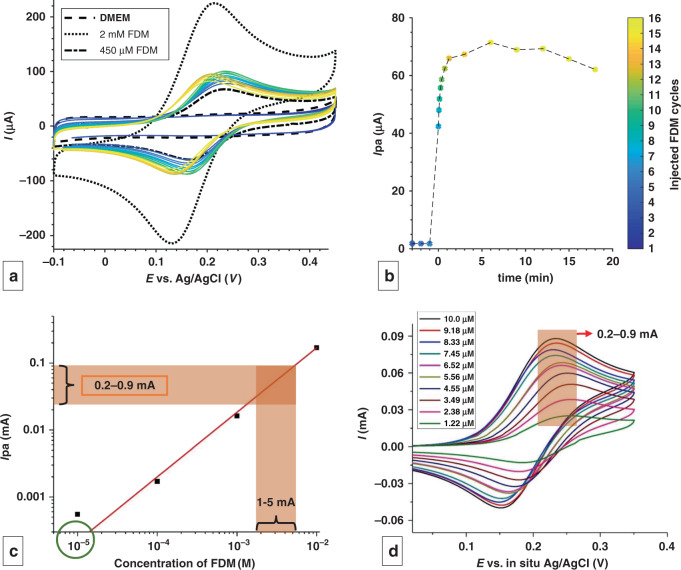
Electrochemical sensing of a model redox molecule through flexible and porous CV electrodes. **a** CV demonstrating the diffusion of FDM through the pores in real time, with peak height proportional to the local FDM concentration. Scan rate = 300 mV/s, 3.7s/cycle. **b** Evolution of anodic peak current (Ipa) vs time. **c** Calibration plot for FDM in DMEM + 10% FBS in the porous CV sensor. The concentration corresponding to the Ipa values measured from **d** is shown by the brown trace in **c**. The experimental bulk concentration in the bottom chamber is marked by the green circle in **c**. **d** CV following the addition of 10 aliquots of 100 μL of 10 mM FDM. The legend displays the final bulk concentration

The 3D-printed cell culture platform with integrated electrochemical sensors on the bottom side of the membrane can be used to measure metabolites or biomarkers released from cells growing on the top side. To test the functionality of the system, we characterized this platform with a well-understood redox molecule, ferrocene dimethanol (FDM). To simulate CV monitoring of metabolite release from a cell layer, 1 mL of 2 mM FDM was injected above the top side of the membrane and allowed to diffuse across the membrane, react at the CV sensor at the bottom side of the membrane, and diffuse into the bottom chamber containing 3.5 mL of DMEM. CV was performed at 300 mV/s in the potential range of −0.1–0.45 V for high time resolution of the diffusion kinetics. The colored solid plots in Fig. 4a show real-time monitoring of the diffusion of FDM in DMEM through the porous membrane, where the anodic peak current is extrapolated over time in Fig. 4b. The plot shows upper and lower bounds of 2 mM and 450 μM, the lowest concentration achievable after the complete dissolution of the injected 2 mM FDM into the full 4.5 mL volume. The actual measured concentration of injected FDM lies between these two bounds, where the cycle number is denoted by a color bar (Fig. 4a, b). The Nernst equation dictates that the ideal behavior of a redox molecule at the electrode occurs when the currents of the anodic peak current (Ipa) and cathodic peak current (Ipc) are equal (Ipa/Ipc ~1), and the difference between the anodic peak potential (Epa) and cathodic peak potential (Epc) is ~59 mV for a one-electron transfer reaction^46^. These conditions are closely met for FDM detection (Ipa/Ipc ~0.8, Epa−Epc = 56 mV), indicating that the three electrodes fabricated on the porous membrane are on par with conventional three-electrode CV systems.

The kinetics of the diffusion of FDM can be seen by plotting Ipa from each cycle vs time in Fig. 4b. Here, the diffusion of FDM across the membrane to the CV electrode is instantaneous, as evidenced from the sudden spike at *t* = 0 (cycle 4) when FDM is added. The anodic current increases rapidly (cycle 4–11) as FDM continues to diffuse across the membrane, followed by a plateau and slow decrease in the signal (after cycle 11) as FDM becomes diluted in the bulk solution of the bottom chamber. These results demonstrate that molecular detection is feasible with an electrode-integrated porous membrane with a temporal resolution of ~1 min in a diffusion-limited manner.

Apart from yielding qualitative molecular sensing, the porous sensor yields quantitative information on metabolite concentration in complex media, such as DMEM + FBS, provided the media is devoid of any redox-active species that undergoes reduction and oxidation in the potential window of the molecule of interest. Figure 4c shows a calibration plot for FDM concentrations measured using membrane-integrated CV electrodes, which shows a linear range from 100 μM to 10 mM (*n* = 3). FDM solutions were measured at constant concentrations equilibrated across the top and bottom chambers to avoid diffusion/transient effects, which resulted in near-Nernstian behavior (Ipa/Ipc ~0.85, Epa−Epc ~70 mV).

Following this electrode calibration, another FDM injection experiment was performed to analyze the difference in concentration measured at the CV sensor location on the membrane (Fig. 4d) vs. the concentration in the bulk (Fig. 4c). FDM (10 mM) in DMEM and FBS was added in aliquots of 100 μL to the empty top chamber in 10 steps over 2 min intervals, as measured in real time by CV. The final expected concentrations, after complete diffusion of FDM in the bottom chamber over the 10 steps, ranged from 1.22 μM to 10 μM, as shown in the legend of Fig. 4d. However, the measured Ipa values just after diffusion across the membrane ranged from 0.2 mA to 0.9 mA (Fig. 4d), which correspond to concentrations in the range of 1–5 mM when compared to the calibration plot (indicated by brown sections in Fig. 4c). This result shows that concentrations measured near the membrane at the start of the diffusion process are nearly three orders of magnitude larger than the anticipated final concentration diluted in the bulk (1.22 μM to 10 μM FDM is indicated by a green circle on Fig. 4c), highlighting the utility of in situ molecular detection.

**Fig. 5:**
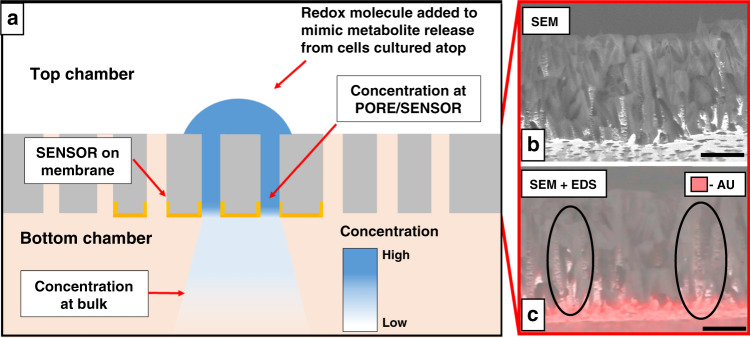
CV detection scheme of molecular diffusion across an electrode-integrated porous membrane. **a** Cross-sectional schematic of the porous sensor and diffusion of FDM. The blue semicircle represents the addition of FDM to the top chamber. The Au working electrode (sensor) fabricated on the bottom side of the porous membrane is shown as yellow caps. The gradient in blue illustrates the diffusion-assisted concentration gradient in the system. **b** The cross-sectional SEM of the membrane shows pore channels in all directions. **c** Cross-sectional SEM + EDS shows the distribution of e-beam-deposited Au (scale bar: 8 μm)

In Fig. 5a, an illustration of transmembrane diffusion is used to explain the benefit of using electrode-integrated porous cell culture membranes for sensing cell-released molecules. Upon adding an aliquot of 10 mM FDM on the top side of the membrane, it diffuses and dilutes along the length of the pore. The cross-sectional SEM and EDS results reveal the geometry and the distribution of Au in the pores, respectively (Fig. 5b). The coupled SEM and EDS results show that Au deposited on the surface can also reach several microns into the pores, specifically if they are oriented perpendicular to the membrane (Fig. 5c). This deposition can enable sensing much closer to the point of injection where FDM is in high concentrations. If given infinite time, 10 mM FDM will diffuse and dilute itself in the entire volume of the bottom chamber. However, as the CV measurements are made on the membrane within 2 min following FDM addition, the measured current level reflects the concentration in the pore adjoining the sensor before the dilution, as shown by the sustained plateau of FDM at its highest concentration between 1 and 10 min after addition (Fig. 4b). The measured concentration will depend on the diffusion coefficient of the molecule in the pores and the time of measurement. Interfacial, membrane-integrated sensors can be advantageous over offline sensors and downstream sensors because these methods lack the capability to sample molecules at the site of release and may result in low or even loss of sensitivity. Interfacing such electrochemical biosensors directly with the tissue culture membrane will facilitate the sensing of small quantities of the key molecules that were previously undetectable by downstream fluidic sensors.

### In situ multimodal impedimetric and electrochemical CV sensors

**Fig. 6:**
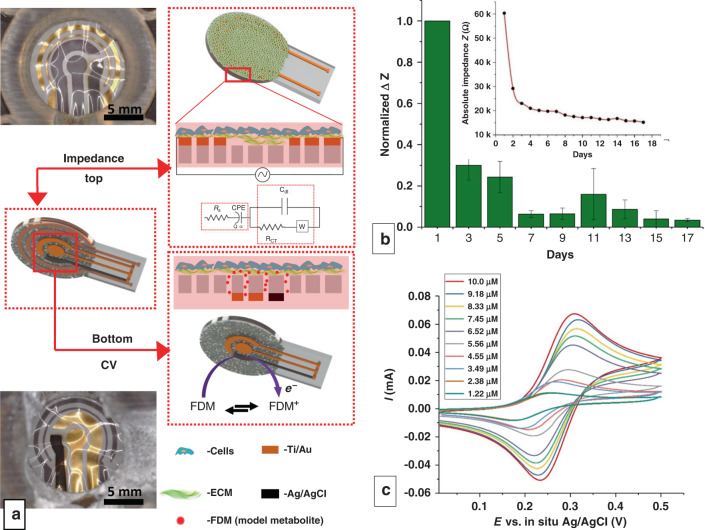
Multimodal sensor-integrated transwell system. **a** Optical images and schematic illustrations of the multimodal sensors displaying both impedimetric (top view) and CV (bottom view) sensors. Electrodes in view are lustrous Au and black Ag/AgCl, while electrodes on the opposite side of the translucent membrane are gray due to the Ti adhesion layer. Membranes appear wrinkled due to thermal expansion during PDMS curing. **b** Normalized change in impedance values measured at 10 Hz over the course of 17 days. (*n* = 3, measurements from 3 separate devices). The inset shows the absolute change in impedance from one of the devices tested. **c** CV following the addition of 10 aliquots of 100 µL of 10 mM FDM on the apical side of the gut triculture. The legend displays the final experimental concentration

In the previous sections, impedimetric and CV sensors were patterned on different membranes and demonstrated separately. These two sensing modalities, if integrated together, can provide simultaneous, high-sensitivity, and high temporal resolution measures of physical and biochemical processes following cellular stimulation and signal transduction. For instance, this approach would provide the crucial ability to correlate loss of gut epithelial barrier integrity with increased or decreased serotonin release, as is implicated in gastrointestinal disorders^57,58^. In case II, a multimodal sensor-integrated membrane was fabricated with concentric impedance electrodes on the top side and CV electrodes on the bottom side and then used to impedimetrically sense cell growth and potentiometrically sense FDM, mimicking metabolite/biomarker release (Fig. 6a). The membrane was seeded with the triculture as described in case I, but new fabrication challenges and details are described in the Supplemental information, including Supplementary Fig. S4. Analogous to the IDEs, the pair of circular impedance electrodes was able to sense the progress of cell culture, showing an exponential drop in impedance as the cell culture progressed (Fig. 6b). The inset shows the absolute values of impedance in one of the trials showing a ~300% drop in impedance, nearly double the impedance drop observed in case I. The difference in normalized impedance between case I and case II may be due to changes in the cell seeding density or growth behavior, although each experiment was performed in triplicate with three different cell cultures while maintaining the same trends, so absolute values should not be compared at face value. However, the major difference in case II is that the counter electrode has a 3x larger surface area than the working electrode, which provides unrestricted counter ion flow within the circuit, so it may be concluded that case II electrodes have higher sensitivity due to their design. These results demonstrate that the impedance electrodes are fully functional in the circular configuration.

To mimic metabolite/biomarker release from the basolateral side of a tissue culture, 10 mM FDM in DMEM + FBS was added to the top chamber just above the cell layer and allowed to diffuse down across the membrane (Fig. 6c), similar to the no-cell case in Fig. 4c, d. FDM (10 mM) was added in aliquots of 100 μL to the top chamber in 10 steps, followed by real-time CV measurements at 2 min intervals. The FDM detection results appear similar to those in the no-cell case, with slightly lower CV peaks accounting for limited diffusion through the cell layer. Future work will compare this result to the measurement of metabolites released from the basolateral side of a cultured cell layer, which would not be limited by diffusion through the cell layer but may still be limited by diffusion through the secreted ECM. Similar CV plots were obtained as before, but the peak potentials were shifted by +0.1 V (Fig. 6c). We hypothesize that this shift may have been due to the long-term exposure (17 days) of the Ag/AgCl reference electrode to the redox environment of DMEM + FBS and cellular effluents, which can impact its standard redox potential due to oxidation or reduction of AgCl. Even after significant exposure and fouling for 17 days, the reference, working, and counter electrodes maintained their functionality, as evidenced by the characteristic CV curves. As explained previously, the current measured by the CV sensors corresponds to the undiffused and undiluted analyte concentrations in the pores near the initial injected concentration. This feature is a significant advantage of this platform, as the cells and the sensors are directly connected through pores in the membrane. These results suggest the ability to detect redox-active cellular metabolites (e.g., serotonin and ascorbic acid) that interact directly with the electrode before further dilution in the bulk. The sensing of non-redox-active cellular metabolites may require modifying the Au electrodes with appropriate receptors or biorecognition elements. Such modification or functionalization of Au electrodes for various metabolite sensing has been extensively studied^59–61^. Thus, the in vitro platform integration of CV sensors in conjunction with impedance sensors may result in enhanced sensitivity and temporal resolution, enabling unprecedented access to physical and molecular information in cultured tissue and further our understanding of cellular behavior and signaling within transwell assays.

## Conclusion

In this work, we have demonstrated a 3D-printed, modular, electrochemical sensor-integrated transwell system for monitoring cellular and molecular events in situ, utilizing simple additive manufacturing techniques such as 3D printing, shadow masking, and PDMS molding. This system is modular, autoclavable and reusable, circumventing conventional microfabrication processes and enabling this platform to serve as an indispensable and facile discovery tool that can be used in lieu of conventional transwell inserts in cellular/molecular biology.

The platform has all the salient features of a commercial transwell along with impedimetric and electrochemical sensing capability, which can potentially provide improved access to information about the cellular and molecular events that cannot be obtained from endpoint or downstream sensors used in conjunction with a conventional transwell. The membrane integrated impedance electrodes allow facile, noninvasive monitoring of cell growth, circumventing conventional TEER methods. The membrane-integrated CV electrodes have the potential to sense three orders of magnitude higher concentrations of metabolites than dilution in the bulk volume, owing to their configuration and proximity to the cells. Real-time information from the cell-interfaced impedance sensor and the membrane-interfaced CV sensor may provide insights into complex biological systems, especially those that involve physical movement or barrier formation alongside molecular signaling. This modular 3D-printed electrochemical electrode-integrated platform will broadly impact the research and development community due to ease of fabrication and integration into standard cell culture SOPs, revealing real-time temporal information of cellular and molecular signaling events that were previously inaccessible.

## Materials and methods

### Sensor-integrated membrane

The electrodes on the membranes are fabricated with e-beam evaporation (20 nm Ti/100 nm Au and 500 nm Ag) on polyester track etched (PETE) membranes (thickness: 11 μm; porosity: 2 × 10^7^ pores/cm^2^; pore radius: 500 nm) through laser-cut shadow masks. Ag/AgCl reference electrodes are fabricated in situ by oxidizing Ag to AgCl via exposure to 50 mM FeCl_3_ for 45 s^62^. For case I, the impedance electrodes are patterned as interdigitated electrodes (IDEs) with a length of 9 mm and a width and spacing of 500 μm. The CV system is designed with a circular working electrode in the center (total exposed surface area: 16.8 mm^2^) surrounded by semicircular reference and counter electrodes with a total width of 7.5 mm (Supplementary Fig. S2). The contact pads have an interpad spacing of 2.54 mm to pair them with standard off-the-shelf contact pins. Within case II, sensors are fabricated on opposite faces of the same membrane. A pair of concentric electrodes with an inner diameter of 9.5 mm and outer diameter of 13 mm act as impedance electrodes. The widths of the inner and outer electrodes are 250 μm and 750 μm, respectively, with a spacing of 750 μm. The CV electrodes are fabricated with an outer diameter of 8.5 mm. The impedance of the CV electrodes is nested within them with a spacing of 1 mm. Parylene deposition is carried out with a Model 2010 system (Specialty Coating Systems, Indianapolis, IN, USA). The completely assembled device can be autoclaved and stored for use in ambient conditions, similar to a commercial transwell.

### Sensor-integrated cell culture housing

The transwell housing was designed in Autodesk® Inventor™ and 3D-printed with an Objet500 Connex3 3D-printer (Stratasys, Eden Prairie, MN, USA) with MED610 photopolymer using PolyJet technology. In situ PDMS gaskets were fabricated by casting and thermally curing a mixture of prepolymer and curing agent at 60 °C for 5 h in a ratio of 10:1 (Sylgard^®^184, Dow Corning, Midland, MI, USA) on the 3D-printed channels. The sensor-integrated cell culture membrane was sandwiched between in situ o-rings between the top and bottom chambers. Metric screws were used to fasten the chambers together. The gasket was coated with a thin film of uncured PDMS before sandwiching the sensor-integrated membrane between the two chambers and thermally cured to seal the membrane in place. This sealing step, in addition to the PDMS gaskets, ensures a leak-free setup. The housing was then autoclaved at 121 °C for 30 min to sterilize before use.

### Cell culture, TEER, and immunocytochemistry

DMEM (Sigma-Aldrich, St. Louis, MO, USA) with 10% FBS (ThermoFisher, Waltham, MA, USA) was used as the cell culture medium. Cells were cultured at 37 °C and 10% CO_2_ in an Isotemp incubator from Fisher Scientific. Caco-2, HT29-MTX, and RIN14B cell lines (ATCC^®^, Manassas, VA, USA) were thawed, passaged once, and seeded onto the 3D-printed device at a ratio of 4:2:1. The cultured cells were harvested from T75 flasks, counted, and mixed to yield a total count of 0.05×10^6^ cells (analogous to the standard protocols for seeding a 12-well plate). The devices seeded with cells were incubated at 37 °C and 10% CO_2_. The medium was exchanged once every 2 days. TEER was measured with an epithelial voltmeter (EVOM2™) using chopstick electrodes through the ports (Supplementary Fig. S1). To fix and stain the cells, the membrane was removed and fixed in 4% paraformaldehyde for 30 min, rinsed three times with 1× phosphate buffered saline (0.1 M PBS pH = 7.4), and stained with 1 mg/mL DAPI (blue) for DNA. For staining tight junctions, the membrane was incubated with 1% bovine serum albumin (BSA) to prevent nonspecific binding, followed by incubation with rabbit anti-rat polyclonal primary antibody against occludin (1:100). The primary antibodies were incubated overnight at 4 °C. The membrane was rinsed in 1× PBS three times and incubated with donkey anti-rabbit IgG (H + L) secondary antibody and Alexa Fluor 488 conjugate (1:100, Invitrogen, Carlsbad, CA, USA). The membrane was imaged with a confocal microscope (Zeiss LSM 700, Oberkochen, Germany) after rinsing three times in 1× PBS.

### Electrochemical measurements

All measurements were carried out in sterile conditions in a biosafety cabinet. A VSP-300 potentiostat (Bio-Logic Inc., Seyssinet Pariset, France) was employed for all electrochemical measurements. Spring-loaded Au pushpins and female header pins (Digi-key Electronics, Thief River Falls, MN, USA) ware used to make electrical contact with the sensors on the platform. Impedimetric measurements were carried out by applying a sinusoidal AC voltage with an amplitude of 10 mV_rms_ at a frequency range of 10 Hz to 10 MHz. Data were analyzed by equivalent circuit modeling with the Z-fit module of EC-lab (Bio-Logic Inc). CV measurements were recorded within a potential window of 0–0.5 V at a scan rate of 100 mV/s, unless otherwise stated. In this study, 10 mM FDM in 0.1 M PBS (pH = 7.4) or DMEM with 10% FBS was used as the redox molecule.

## Supplementary information

Supplemental Materials
