# Supplementary material for: 3D-Printed electrochemical sensor-integrated transwell systems
Source: Microsyst Nanoeng. 2020 Oct 5;6:100. doi: 10.1038/s41378-020-00208-z (PMC8433167; doi:10.1038/s41378-020-00208-z)
Supplement: Supplementary file 1 — Supplemental Materials [file 41378_2020_208_MOESM1_ESM.pdf]

3D-PRINTED ELECTROCHEMICAL SENSOR-INTEGRATED TRANSWELL SYSTEMS

P. Ramiah Rajasekaran[1], *A. Chapin*[2], *D.N. Quan*[2], *J. Herberholz*[5],

W.E. Bentley[2,3,4], *and R. Ghodssi*[1,2,4,6*]

[1]Institute for Systems Research, [2]Fischell Department of Bioengineering, [3]Institute for Bioscience and Biotechnology Research, [4]Robert E. Fischell Institute for Biomedical Devices, [5]Department of Psychology and Neuroscience and Cognitive Science Program, [6]Department of Electrical and Computer Engineering,

University of Maryland, College Park, Maryland, USA

Supplemental Information

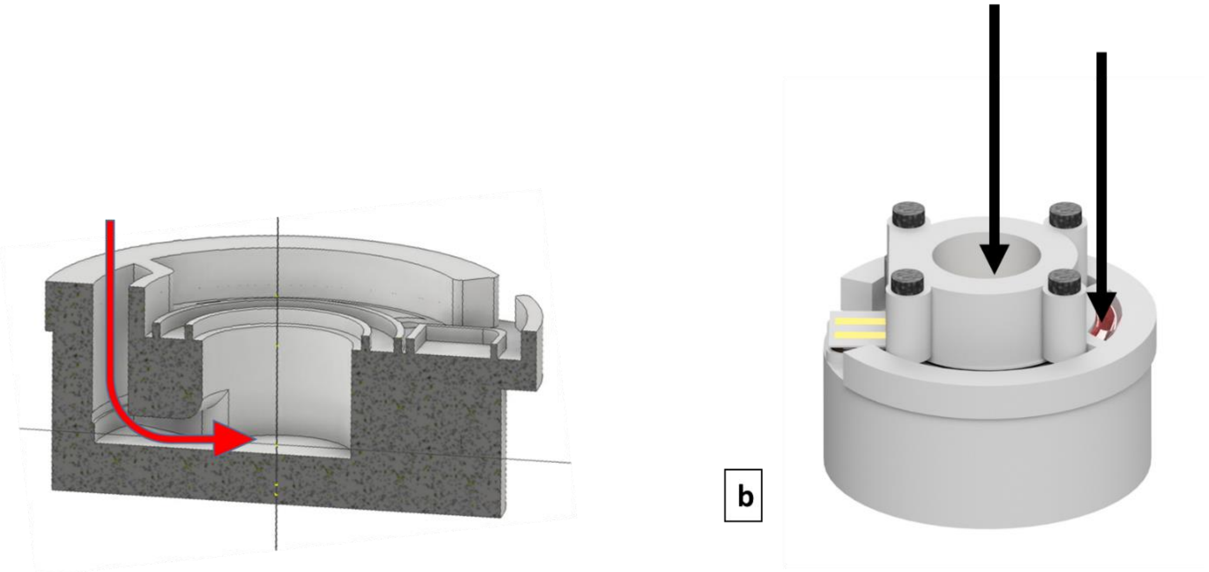

a

b

Figure S1. a) Cross-sectional view of the device showing the internal connection between the raised access port and the bottom chamber (red arrow). b) The schematic of the 3D-printed sensor integrated platform without the lid. The black arrows indicate area for the placement of conventional chop stick TEER electrodes.

Modeling

To further validate our experimental results, the Z-fit module from EC-lab was used to model and fit an equivalent circuit that corresponds to our system (Fig 3). The platform consists of a porous polymeric membrane with gold electrodes in direct contact with the cell culture media containing dissolved electrolytes and proteins. Constant phase element (CPE) is the preferred circuit element for modeling a porous electrode-electrolyte interface, which is analogous to our system.[1] R_s represents the solution resistance. CPE confers a linear correlation between the real and imaginary impedance values, which also imparts a diffusion-limited pattern to the system (Fig 3a). Randle's circuit is the preferred circuit used to model an electrode-tissue interface, and is therefore used to model the interface between the cell culture and our electrode.[2] Randle's circuit includes a charge transfer resistance (R_{CT}) and a double layer capacitance (C_{dl}) in parallel, along with a Warburg diffusion element (W), the latter of which is characterized by a semicircular head followed by a linear tail (Fig 3b). Therefore, the equivalent circuit of the electrode-media-cell

culture interface is modeled with R_s, CPE, and Randle's circuit in series. It can be used to model both the control with electrode and media, and the platform with electrode, media, and cells, as seen from Fig 3c and 3d, where the dots represent the experimental data and the solid line corresponds to the model. A close fit between the experimental (dots: Z) and simulated data (lines: Z fit) shows that this simple model can divulge information about the electrical properties of the system. The model gives the values of R_s (solution resistance) to be approximately between 25 – 30Ω, which corresponds to the resistance of DMEM. Figure 2d shows the emergence of R_{CT}, which can also be obtained from the model. The control and the first two days of the cell culture generated insignificant values for charge transfer resistance (R_{CT}), which can be seen from the absence of the characteristic semicircular feature. As the cell culture progresses, the R_{CT} values from the model show a transition from negative to positive, followed by a plateau at 10Ω. This increase in charge transfer resistance can be attributed to the surface coverage by growing tissue and ECM, limiting charge transfer between the media and the electrode, which can be qualitatively related to the barrier integrity of the cell culture. This shows that the cell-interfaced porous impedance sensor embedded in the 3D-printed platform can be used to qualitatively monitor cells and tissues. Previously, electrode-integrated surfaces have been used to impedimetrically monitor cell culture.[3,4] However, this work is the first demonstration of impedimetric sensing of cell growth on a porous and flexible membrane-electrode-cell interface. As this cell culture sensing platform can be fabricated with simple additive manufacturing tools and follows the SOP of a commercial Transwell® assays, it can be used to non-invasively monitor cell/tissue culture routinely in a laboratory setting. A side-by-side comparison of the Transwell® with the sensor-integrated 3D-printed transwell shows that this modular platform has all the features and accessibilities of a Transwell® with the added advantage of real-time multimodal sensing capability (Fig 1).

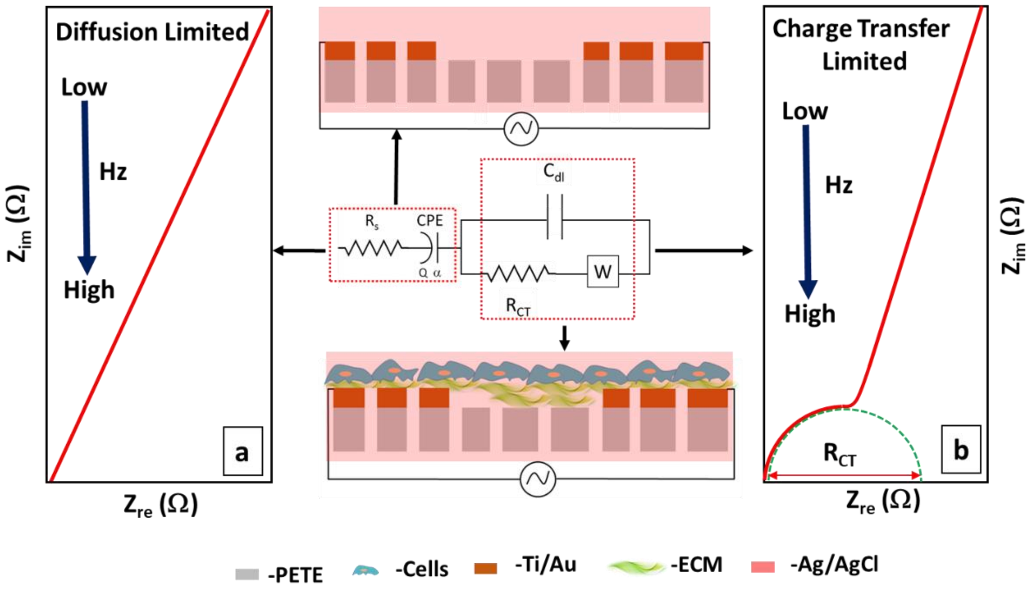

Figure S2. Equivalent circuit model for the cell-covered porous electrode. a) Nyquist plot of the bare electrode showing characteristics of a diffusion-limited process, in the absence of cells (middle, top). b) Nyquist plot when the system impedance is governed by the charge transfer resistance (R_{CT}) upon full coverage by cells and ECM (middle, bottom). The corresponding model is shown at the center. R_s, Q, α, C_{dl} and W are solution resistance, constant phase element (CPE) parameter, CPE exponent, double layer capacitance, and Warburg element, respectively.

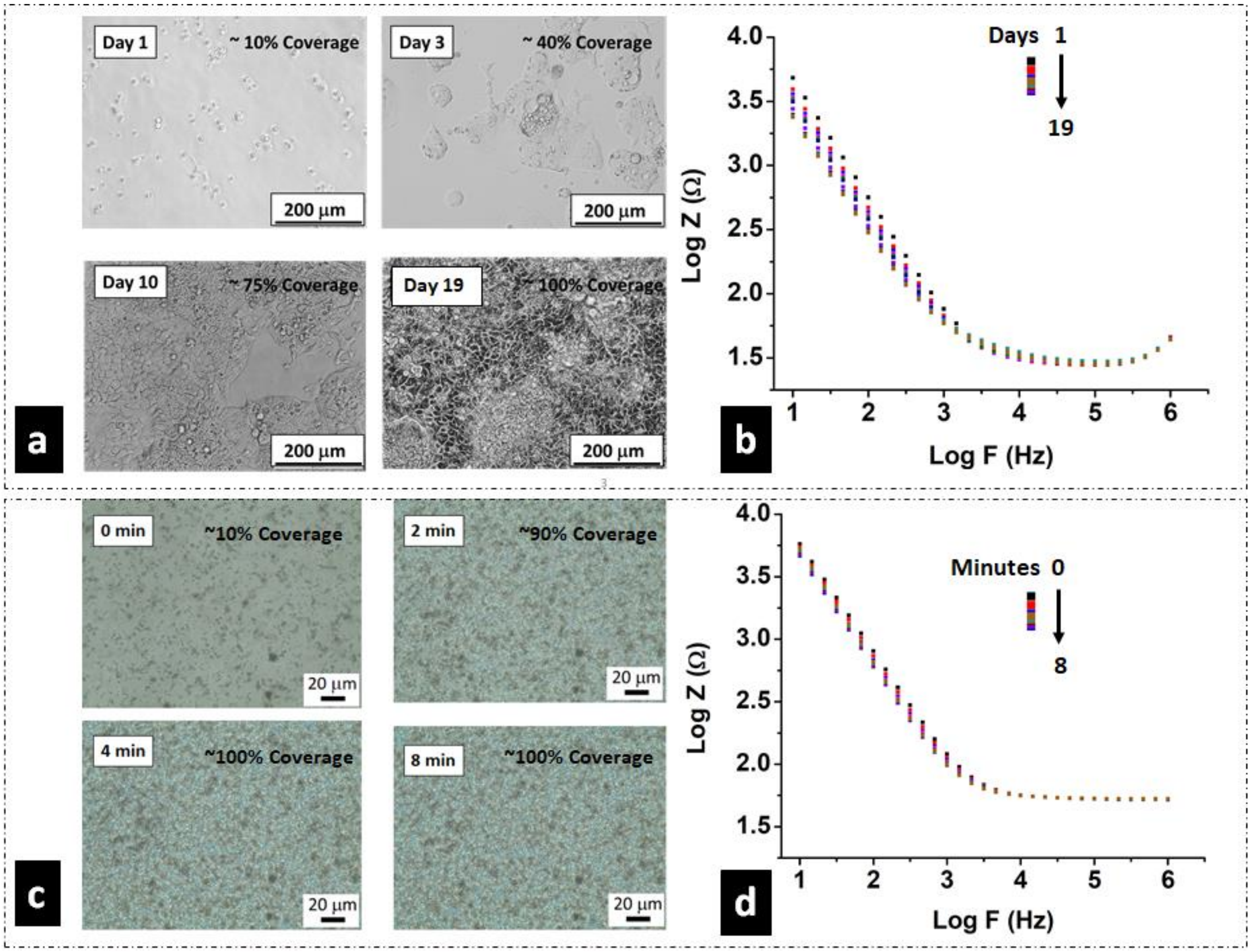

Figure S3. Optical and impedimetric detection of cell coverage (a,b) and alumina nanoparticle coverage (c,d). a) Optical images of cell coverage in a 6 well plate over a time period of 19 days (~10% to 100% surface coverage). b) Impedance measurements recorded from the 3D-printed device plated with cells in identical conditions. c) Optical images of deposition and coverage of alumina microparticles dispersed in cell culture media on a glass slide over a time period of 8min (~10% to 100% surface coverage). d) Impedance measurements recorded from the 3D-printed device with same quantity of alumina under identical conditions. The real impedance shows a ~ 68% decrease during cell coverage and a ~7% decrease during coverage with alumina.

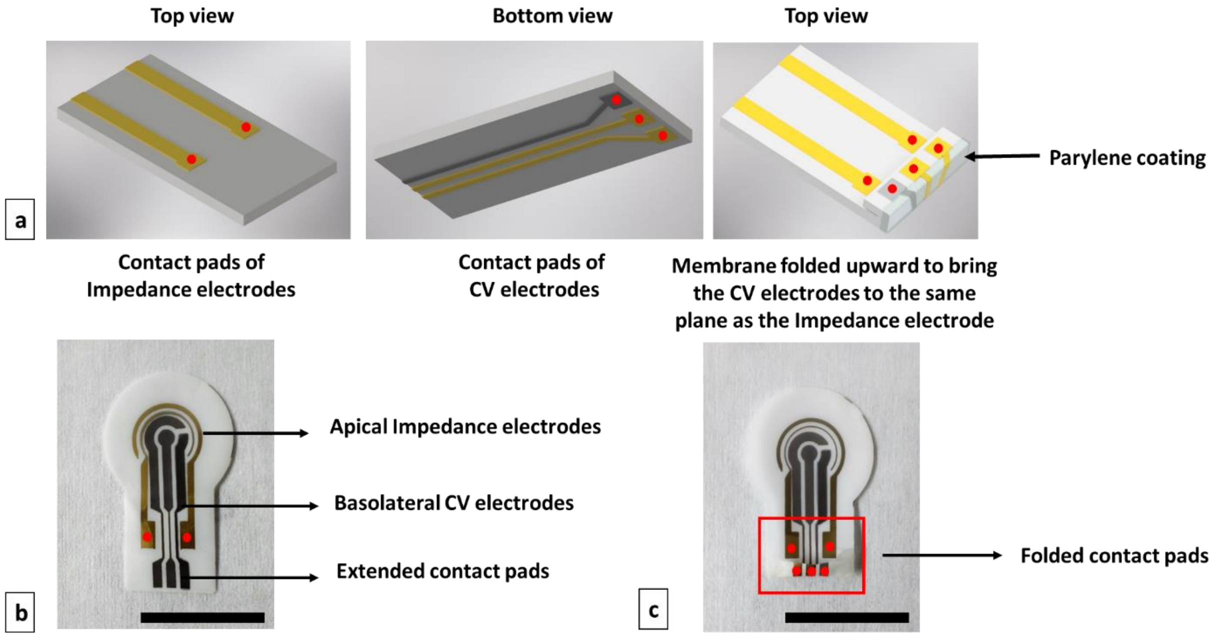

Figure S4. a) Schematic of the strategy used to fold the membrane to orient the CV electrode contact pads face-up. b) Top view image of the multimodal electrode-integrated membrane, showing the geometrical arrangement of the two sensors and the extended contact pad that will be folded. c) Image of the parylene-coated contact pads folded to bring all the contact pads onto the same face. (scale bar: 15mm). Red dots indicate the point of contact for sensing. The membrane is folded post-fabrication to accommodate all the contacts on one plane such that sensing from both impedance and CV electrodes can be performed by contact from one set of contact pins.

Fabrication Challenges Associated with Multimodal Sensor Integration

Two new challenges are introduced upon integration of both electrochemical sensing modalities on the same membrane, making contacts to both sides of the membrane and avoiding electrical crosstalk. The first challenge is addressed by folding back the CV electrode contact pads so that they face upwards and can be connected to contact pins alongside the impedance electrode contact pads, as described in Supplementary Figure S4. The area to be folded is reinforced with parylene to prevent the electrodes from fracturing during bending. The second challenge requires that the two sensors be used in a staggered fashion, whereas significant noise would be introduced if measurements were taken in sync.

In both case I and II, PDMS is cured around the membrane edge to seal it inside the 3D-printed platform. Thermal treatment in the autoclave causes reversible thermal expansion of the 3D-printed device, and the device seals the membrane in place in an expanded state. Upon cooling down, the 3D-printed structure shrinks, compressing the membrane to form wrinkles. The non-planar nature of the membrane is evidenced by the diffuse reflective patterns observed from optical images of both the top and bottom face of the membrane (Fig. 6a).[5] The wrinkled, non-flat landscape of the membrane is similar to the macroscopic mm-scale folds of the intestinal landscape.[6] This non-planar architecture may be used to impart a biomimetic character to the sensor-integrated transwell membrane.

REFERENCES:

(1) Koklu, A.; Sabuncu, A. C.; Beskok, A. Rough Gold Electrodes for Decreasing Impedance at the Electrolyte/Electrode Interface. *Electrochim. Acta* **2016**, *205*, 215–225. https://doi.org/10.1016/j.electacta.2016.04.048.

(2) Ovadia, M.; Zavitz, D. H. The Electrode – Tissue Interface in Living Heart : Equivalent Circuit as a Function of Surface Area. *Electroanalysis* **1998**, *10* (4), 262–272.

(3) Lukic, S.; Wegener, J. Impedimetric Monitoring of Cell-Based Assays. In *eLS*; 2015; pp 1–8. https://doi.org/10.1002/9780470015902.a0025710.

(4) Lei, K. F. Review on Impedance Detection of Cellular Responses in Micro/Nano Environment. *Micromachines* **2014**, *5* (1), 1–12. https://doi.org/10.3390/mi5010001.

(5) van Ginneken, B.; Stavridi, M.; Koenderink, J. J. Diffuse and Specular Reflectance from Rough Surfaces. *Appl. Opt.* **1998**, *37*, 130–139. https://doi.org/10.1364/AO.37.000130.

(6) Koppes, A. N.; Kamath, M.; Pfluger, C. A.; Burkey, D. D.; Dokmeci, M.; Wang, L.; Carrier, R. L. Complex, Multi-Scale Small Intestinal Topography Replicated in Cellular Growth Substrates Fabricated via Chemical Vapor Deposition of Parylene C. *Biofabrication* **2016**, *8* (3). https://doi.org/10.1088/1758-5090/8/3/035011.
